# Protocol paper for the ‘Harnessing resources from the internet to maximise outcomes from GP consultations (HaRI)’ study: a mixed qualitative methods study

**DOI:** 10.1136/bmjopen-2018-024188

**Published:** 2018-08-10

**Authors:** Maureen Seguin, Laura Hall, Helen Atherton, Rebecca Barnes, Geraldine Leydon, Elizabeth Murray, Catherine Pope, Sue Ziebland, Fiona A Stevenson

**Affiliations:** 1 eHealth Unit, Primary Care and Population Health, University College London, London, UK; 2 Centre for Global Chronic Conditions, London School of Hygiene and Tropical Medicine, London, UK; 3 Unit of Academic Primary Care, Warwick Medical School, University of Warwick, Coventry, West Midlands, UK; 4 Centre for Academic Primary Care, Population Health Sciences, Bristol Medical School, University of Bristol, Bristol, UK; 5 Primary Care and Population Science, Faculty of Medicine, University of Southampton, Southampton, UK; 6 Health Sciences, University of Southampton, Southampton, UK; 7 Nuffield Department of Primary Care Health Sciences, University of Oxford, Oxford, UK

**Keywords:** primary health care, general practice consultations, family practice, physician-patient relations, video-recording conversation analysis

## Abstract

**Introduction:**

Many patients now turn to the internet as a resource for healthcare information and advice. However, patients’ use of the internet to manage their health has been positioned as a potential source of strain on the doctor–patient relationship in primary care. The current evidence about what happens when internet-derived health information is introduced during consultations has relied on qualitative data derived from interview or questionnaire studies. The ‘Harnessing resources from the internet to maximise outcomes from GP consultations (HaRI)’ study combines questionnaire, interview and video-recorded consultation data to address this issue more fully.

**Methods and analysis:**

Three data collection methods are employed: preconsultation patient questionnaires, video-recorded consultations between general practitioners (GP) and patients, and semistructured interviews with GPs and patients. We seek to recruit 10 GPs practising in Southeast England. We aim to collect up to 30 patient questionnaires and video-recorded consultations per GP, yielding up to 300. Up to 30 patients (approximately three per participating GP) will be selected for interviews sampled for a wide range of sociodemographic characteristics, and a variety of ways the use of, or information from, the internet was present or absent during their consultation. We will interview all 10 participating GPs about their views of online health information, reflecting on their own usage of online information during consultations and their patients’ references to online health information. Descriptive, conversation and thematic analysis will be used respectively for the patient questionnaires, video-recorded consultations and interviews.

**Ethics and dissemination:**

Ethical approval has been granted by the London–Camden & Kings Cross Research Ethics Committee. Alongside journal publications, dissemination activities include the creation of a toolkit to be shared with patients and doctors, to guide discussions of material from the internet in consultations.

**Strengths and limitations of this study**

- A key strength of the study is the use of mixed qualitative methods used to capture what happens during consultations.
- This study will be the first empirically based indication of how conversations on online health information occur in general practice.
- The findings of this study will inform the first ever empirically based toolkit for patients and general practitioners to help guide discussion of online health information, minimising interactional disruption during consultations.
- We may yield a low number of recorded consults wherein the internet is mentioned, thereby limiting what we can explore and conclude about such conversations.

## Background

Increased access to the internet has provided patients with access to previously privileged information such as health information aimed at health professionals, alongside experiential information shared by other patients.^1^ These developments have heightened the pre-existing ‘double bind’ for patients, who are supposed to be knowledgeable about health conditions and able to manage their own care, yet defer to medical wisdom in the consultation itself.^2^

Previous research has established that both patients and doctors are aware of the tension experienced by patients resulting from expectations to be both knowledgeable and passive during the consultation.^3^ Though patients are making wide use of the internet for health information, many have concerns about sharing this with their doctors, fearing to be seen as challenging medical authority.^4 5^ Those who do initiate discussions about internet-derived health expect to have the information, and their efforts at self-management, taken seriously.^6^ Many general practitioners (GP) report concerns about how to respond appropriately when patients refer to such information in consultations, especially considering the limited time available in typical medical consultations for extended discussions.^7^ They may also be concerned that discussions about internet-derived health information will encourage patients to ‘go online,’ which may lead to undue patient anxiety. Unfortunately, even occasional miscommunications between GPs and patients can have serious deleterious effects, including loss of patient trust and breakdown of the doctor–patient relationship.^6^

The existing evidence base on consultation discussions of internet-derived health information relies on self-reports, either from interview or questionnaire studies with patients and/or GPs. However, these approaches are vulnerable to the criticism that self-report post hoc accounts reflect respondent reconstructed accounts of what happened. Alternatively, consultation recordings provide access to interactional details in consultations which do not rely on accounts from GPs or patients. The ‘Harnessing resources from the internet to maximise outcomes from GP consultations (HaRI)’ study fills a gap in the research by video-recording consultations, in addition to collecting preconsultation patient data via questionnaire and retrospective data via GP and patient interviews after consultation.

The HaRI study aims to establish how material from the internet is managed by GPs and patients in consultations. Our objectives are fivefold: (1) to determine the range of sources of information and advice used by a sample of patients in advance of their appointment; (2) to understand the range of ways in which patients mention, or avoid mentioning, prior or future use of the internet and how GPs respond and/or refer to the internet themselves; (3) to explore patients’ perspectives on their consultation and reflect on how easy or otherwise it was to discuss prior and planned use of the internet as well as their views about, and experiences of, GPs looking up material online during the consultation and GP recommendations for the use of online material; (4) to explore GPs’ perspectives on the introduction/management of material from the internet by patients in the consultation and their views about, and experiences of, looking up material online during the consultation and recommendations for patients’ future use; and (5) to synthesise the data from objectives 2, 3 and 4 to provide (1) examples of effective consultation practice for GPs, and (2) guidance for patients on raising the topic of the internet with their GP.

## Methods and study design

The methodological approach for the HaRI study builds on a previous study of doctor–patient communication about medicines in general practice.^8^ It consists of three elements: preconsultation questionnaires, video-recorded consultations and semistructured interviews with GPs and patients. We will recruit 10 GPs working in practices situated in Southeast England, purposively sampled for practice size and whether they are a training practice to capture different patient populations and GP communication behaviours. We will aim to recruit an equal number of male and female GPs, representing a variety of ages and ethnicities. To maximise cost efficiency of fieldwork up to two GPs from a given practice may be involved in the study. We will draw on our local Primary Care Research Networks and Noclor research support, a research service which supports projects based in northern London, to assist with the identification of suitable practices and recruitment of GPs.

## Preconsultation questionnaires and video-recorded consultations

We aim to collect 300 preconsultation questionnaires and associated video-recorded consultations (30 consultations per GP). Recent research places recruitment rates for consultation video recording at 79%,^9^ indicating reasonable acceptability. Patients will be alerted to the study either when booking their appointment by telephone, or by GP reception staff on presentation at their GP surgery. In both cases, they will be handed a brief information sheet about the study when they check in for their appointment with their GP. This will alert a HaRI Research Associate (RA) in the waiting room that a potential participant has arrived. The RA will approach the patient to distribute the Patient Information Sheet and discuss the study. Patients who agree to take part will provide informed consent via a paper consent form. Those who decline to take part will be logged, along with the reason if provided. Those who agree to take part will be given a green sheet of paper with a unique participant number printed on the front of it, which indicates to the GP to record the consultation. Those who decline to take part will be provided with a red sheet of paper, communicating to the GP that the patient has not consented to being recorded.

Each consented participant will be invited to complete the preconsultation questionnaire before their consultation. As the questionnaire is very brief, impact on the flow of clinical practice is expected to be minimal. The questionnaire will focus on patients’ use of information sources before the consultation (deliberately not focusing on the internet so as to avoid drawing attention to our particular interest and thereby avoiding a priming effect). Participants will be invited to give consent to be contacted for a semistructured interview following their consultation. The consent forms provide participants with the option to consent to the recordings and forms to be stored in the University College London (UCL) data archive for use in future research, subject to an appropriate protocol and ethical approval.

The preconsultation data will largely be collected via tablets using Research Electronic Data Capture (REDCap). This method may be problematic for people unfamiliar with tablet computers, or for those who have impaired fine motor control. The researcher will assist and provide paper copies as necessary, which will then be manually entered into REDCap by the RA following data collection.

## Semistructured patient and GP interviews

Our sample of up to 300 consultations and associated questionnaires will provide maximum variation from which to purposively select up to 30 patients (approximately three per GP) for semistructured interviews, aiming for a spread of patient characteristics and experiences. Patients will be selected to reflect a wide range of sociodemographic characteristics and use and reference to internet-derived health information. On the latter, the interview participants will be selected to reflect (1) instances in which patients report consulting the internet before their GP consultation and this is raised in the consultation; (2) instances in which patients report consulting the internet before their GP consultation and this is not raised in the consultation; (3) instances in which the patient refers to the internet in the consultation and this is not reported in the preconsultation questionnaire; and (4) instances in which the GP uses the internet or raises internet use during the consultation. Interviews will explore patient accounts of whether they accessed information on their health issue prior to coming in to see their GP, including such sources as magazine and/or newspaper articles, television programmes or advice from friends and family. We will also explore whether they sought online health information before the consultation, perceptions of GP usage of internet resources during consultations and discussions of internet-derived health information in the recorded consultation.

In addition to patient interviews, we will interview all 10 participating GPs. This will enable the research team to access GPs’ views of patient internet usage to access health information, their own usage of internet resources during consultations and their perceptions of the discussions with patients on online health information. All interviews will be arranged at a time and place convenient to the participating patient or GP. It will be stressed they are free to withhold response to interview questions or to terminate the interview if they wish. Audio interview data will be saved on an encrypted hard drive and transferred into the UCL data safe haven at the first opportunity.

## Ethical considerations

Ethical approval was granted by the London–Camden & Kings Cross Research Ethics Committee on 8 August 2016. We will include consultations with children and those in which a third party aids an adult patient either due to a language barrier or to provide support, so long as the patient is judged by the researcher to be able to give informed consent. Separate consent forms and Patient Information Sheets for children aged 4–10 and 11–17, as well as for adult companions to patients will be used. We will exclude potential respondents unable to consent due to cognitive impairment. Names will be recorded on consent forms and contact details will be collected if participants opt to receive a summary of the findings at the end of the project. These will be kept in a locked filing cabinet, accessible only to the research team. Contact details will be shredded after the reports are sent. Identifying details of participants will be removed at the point of transcription. Video data remain identifiable but will only be seen outside of the project team following explicit informed consent from patients and GPs. The consent forms for the preconsultation questionnaires and consultation video records provide participants with an option for their data to be stored in the UCL data archive to allow their data to be used in future research, subject to an appropriate protocol and ethical approval.

The REDCap platform, which will be used to collect the preconsultation questionnaires, is a secure, web-based application hosted by UCL designed to enable responses to be automatically uploaded into UCL’s data safe haven to maximise data security. The data safe haven has been certified to the ISO 27001 information security standard and conforms to the National Health Service Information Governance Toolkit. It was created using a ‘walled garden’ approach, where the data are stored, processed and managed within the security of the system, avoiding the complexity of assured endpoint encryption. A file transfer mechanism enables information to be transferred into the data safe haven simply and securely. Data from preconsultation questionnaires will be analysed in the data safe haven. As the data safe haven is not currently able to manage video files, the video-recorded data will be stored on an encrypted hard drive.

Requesting the completion of preconsultation questionnaires and recording of consultations is potentially intrusive. It will be made clear that the decision to participate or decline will not affect clinical care and that patients can withdraw consent up to the time all the data are collected at their GP’s surgery. Patients will be given contact details for the research team, including an email address so they can withdraw without having to speak to anyone. GPs will also be made aware that they are free to decline participation and to withdraw consent up until data collection is completed at their site.

## Patient and public involvement

As a team we have extensive experience of working with patient and public involvement (PPI) representatives. Two PPI representatives have assisted the project team in helping shape the research proposal, with particular advice given on the importance and relevance of the research question and the acceptability of the research design for patients. They will also contribute to the design of data collection tools (including the preconsultation survey and interview topic guides), participant information sheets and consent forms to ensure comprehensibility. PPI representatives will be asked to comment on the coding scheme for the analysis and on the interpretation of the data

## Analysis

Descriptive statistics will be conducted on the data yielded by the preconsultation questionnaires, giving a demographic overview of our sample. We will use conversation analysis, a well-established microanalytic approach for the analysis of social interaction,^9^ to analyse vocal and other visible conduct between GPs and patients during consultations. We will screen the data to build collections of cases where patients introduce prior use of the internet to access health information, and where the GP uses or mentions the internet. Cases will be transcribed in detail using the Jeffersonian transcription system to capture what participants say and how they say it, as well as overlap and gaps in talk^10^ to facilitate detailed interactional analysis. Interview data will be analysed using thematic analysis,^11^ and will focus on patients and GPs’ accounts of use of the internet to access health information both in and outside of the consultation. We will follow standard approaches to thematic analysis including familiarisation with data, generating initial codes, searching for and reviewing themes, and defining and naming themes,^11^ thereby safeguarding rigour and avoiding premature formation of themes.

The results will be disseminated via academic routes as well as via professional magazines aimed at GPs and the wider media. We will also seek PPI advice on how and where to disseminate our findings beyond peer-reviewed articles, in particular the development of guidance to support patients wishing to discuss health information derived from the internet in consultations.

## Discussion

This project seeks to investigate a long-standing source of potential miscommunication in general practice, namely discussion in the consultation of the use of the internet. Online health discussions in medical consultations have been publicised recently by Professor Helen Stokes-Lampard, Chair of the Royal College of General Practitioners, who reported that 80% of her consultations are with patients who have already searched online for a diagnosis.^12^ If this is indeed a major source of trouble then we need to describe and delineate the interactional consequences by collecting and analysing consultation recordings, and to improve the evidence base on GP and patient views of online health information. Communication strategies that aid smooth interaction in relation to discussion of the internet can then be identified. These can form the basis of guidance for GPs on how to implement these strategies as well as joined-up guidance for patients on how to effectively raise the topic of the internet with their GP.

## Supplementary Material

Reviewer comments

Author's manuscript
