# Supplementary material for: Protocol paper for the ‘Harnessing resources from the internet to maximise outcomes from GP consultations (HaRI)’ study: a mixed qualitative methods study
Source: BMJ Open. 2018 Aug 10;8(8):e024188. doi: 10.1136/bmjopen-2018-024188 (PMC6089307; doi:10.1136/bmjopen-2018-024188)
Supplement: Author's manuscript [file bmjopen-2018-024188.draft_revisions.pdf]

BMJ Open is committed to open peer review. As part of this commitment we make the peer review history of every article we publish publicly available.

When an article is published we post the peer reviewers' comments and the authors' responses online. We also post the versions of the paper that were used during peer review. These are the versions that the peer review comments apply to.

The versions of the paper that follow are the versions that were submitted during the peer review process. They are not the versions of record or the final published versions. They should not be cited or distributed as the published version of this manuscript.

BMJ Open is an open access journal and the full, final, typeset and author-corrected version of record of the manuscript is available on our site with no access controls, subscription charges or pay-per-view fees (http://bmjopen.bmj.com).

If you have any questions on BMJ Open's open peer review process please email info.bmjopen@bmj.com

BMJ Open

Protocol paper for the ‘Harnessing resources from the internet to maximise outcomes from GP consultations (HaRI)’ study: A mixed qualitative-methods study

Journal:	*BMJ Open*
Manuscript ID	bmjopen-2018-024188
Article Type:	Protocol
Date Submitted by the Author:	14-May-2018

Complete List of Authors:	Seguin, Maureen; University College London, eHealth Unit, Primary Care and Population Health; London School of Hygiene and Tropical Medicine, Centre for Global Chronic Conditions<br>Hall, Laura; University College London, eHealth Unit, Primary Care and Population Health<br>Atherton, Helen; University of Warwick, Unit of Academic Primary Care, Warwick Medical School<br>Barnes, Rebecca; University of Bristol, Centre for Academic Primary Care, Population Health Sciences, Bristol Medical School<br>Leydon, Geraldine; Southampton University, Primary Care and Population Science, Faculty of Medicine<br>Murray, Elizabeth; University College London, eHealth Unit, Primary Care and Population Health<br>Pope, Catherine; University of Southampton, Health Sciences<br>Ziebland, Sue; University of Oxford, Nuffield Department of Primary Care Health Sciences<br>Stevenson, Fiona; University College London, eHealth Unit, Primary Care and Population Health

Keywords:	Primary Health Care, General Practice Consultations, Family Practice, Physician-patient relations, Video-recording, Conversation analysis

SCHOLARONE™
Manuscripts

Title: Protocol paper for the 'Harnessing resources from the internet to maximise outcomes from GP consultations (HaRI)' study: A mixed qualitative-methods study

Corresponding author:

Seguin, Maureen, eHealth Unit, Primary Care and Population Health, University College London, London, UK, and Centre for Global Chronic Conditions, London School of Hygiene and Tropical Medicine, London, UK

Full names, departments, institutions, city and country of all co-authors:

Hall, Laura, eHealth Unit, Primary Care and Population Health, University College London, London, UK

Atherton, Helen, Unit of Academic Primary Care, Warwick Medical School, University of Warwick, Coventry, UK

Barnes, Rebecca, Centre for Academic Primary Care, Population Health Sciences, Bristol Medical School, University of Bristol, UK

Leydon, Geraldine, Primary Care and Population Science, Faculty of Medicine, Southampton University, Southampton, UK

Murray, Elizabeth, eHealth Unit, Primary Care and Population Health, University College London, London, UK

Pope, Catherine, Health Sciences, University of Southampton, Southampton, UK

Ziebland, Sue, Nuffield Department of Primary Care Health Sciences, University of Oxford

Stevenson, Fiona, eHealth Unit, Primary Care and Population Health, University College London, London, UK

Keywords:

Primary health care, general practice consultations, family practice, physician-patient relations, video recording, conversation analysis

Word count excluding title page, abstract, references, figures and tables: 2929

Reference count: 12

ABBREVIATIONS

CA Conversation analysis, GP General Practitioner, HaRI Harnessing resources from the internet, PI Principle Investigator, RA Research Associate, PIS Participant Information Sheet, PPI Patient and Public Involvement, REC Research Ethics Committee, UCL University College London

ABSTRACT

Introduction: Many patients now turn to the internet as a resource for health-care information and advice. However, patients' use of the internet to manage their health has been positioned as a potential source of strain on the doctor-patient relationship in primary care. The current evidence about what happens when internet-derived health information is introduced during consultations has relied on qualitative data derived from interview or questionnaire studies. The 'Harnessing resources from the

For peer review only

For peer review only - http://bmjopen.bmj.com/site/about/guidelines.xhtml

internet to maximise outcomes from GP consultations (HaRI)' study combines questionnaire, interview and video-recorded consultation data to address this issue more fully.

Methods and Analysis: Three data collection methods are employed: pre-consultation patient questionnaires, video-recorded consultations between GPs and patients, and semi-structured interviews with GPs and patients. We seek to recruit 10 GPs practicing in Southeast England. We aim to collect up to 30 patient questionnaires and video-recorded consultations per GP, yielding up to 300. Up to thirty patients (approximately 3 per participating GP) will be selected for interviews sampled for a wide range of socio-demographic characteristics, and a variety of ways the use of, or information from, the internet was present or absent during their consultation. We will interview all 10 participating GPs about their views of online health information; reflecting on their own usage of online information during consultations and their patients' references to online health information. Descriptive, conversation, and thematic analysis will be used respectively for the patient questionnaires, video-recorded consultations, and interviews.

Ethics and Dissemination: Ethical approval has been granted by the London – Camden & Kings Cross Research Ethics Committee. Alongside journal publications, dissemination activities include the creation of a toolkit to be shared with patients and doctors, to guide discussions of material from the internet in consultations.

STRENGTHS AND LIMITATIONS

- A key strength of the study is the use of mixed qualitative methods used to capture what happens during consultations.
- This study will be the first empirically-based indication of how conversations on online health information occurs in general practice.
- The findings of this study will inform the first ever empirically based toolkit for patients and general practitioners to help guide discussion of online health information, minimizing interactional disruption during consultations.
- We may yield a low number of recorded consults wherein the internet is mentioned, thereby limiting what we can explore and conclude about such conversations.

BACKGROUND

Increased access to the Internet has provided patients with access to previously privileged information such as health information aimed at health professionals, alongside experiential information shared by other patients.[1] These developments have heightened the pre-existing "double bind" for patients, who are supposed to be knowledgeable about health conditions and able to manage their own care, yet defer to medical wisdom in the consultation itself.[2]

Previous research has established that both patients and doctors are aware of the tension experienced by patients resulting from expectations to be both knowledgeable and passive during the consultation.[3] Though patients are making wide use of the internet for health information, many have concerns about sharing this with their doctors, fearing to be seen as challenging medical authority.[4, 5] Those who do initiate discussions about internet-derived health expect to have the information, and their efforts at self-management, taken seriously.[6] Many general practitioners (GPs) report concerns about how to respond appropriately when patients refer to such information in consultations, especially considering the limited time available in typical medical consultations for extended discussions.[7] They may also be

For peer review only - http://bmjopen.bmj.com/site/about/guidelines.xhtml

concerned that discussions about internet-derived health information will encourage patients to 'go online,' which may lead to undue patient anxiety. Unfortunately, even occasional miscommunications between GPs and patients can have serious deleterious effects, including loss of patient trust and breakdown of the doctor-patient relationship.[6]

The existing evidence base on consultation discussions of internet-derived health information relies on self-reports, either from interview or questionnaire studies with patients and/or GPs. However, these approaches are vulnerable to the criticism that self-report post-hoc accounts reflect respondent reconstructed accounts of what happened. Alternatively, consultation recordings provide access to interactional details in consultations which do not rely on accounts from GPs or patients. The 'Harnessing resources from the internet to maximise outcomes from GP consultations' (HaRI) study fills a gap in the research by video-recording consultations, in addition to collecting pre-consultation patient data via questionnaire and retrospective data via GP and patient interviews post-consultation.

The HaRI study aims to establish how material from the internet is managed by GPs and patients in consultations. Our objectives are five-fold: (1) To determine the range of sources of information and advice used by a sample of patients in advance of their appointment, (2) To understand the range of ways in which patients mention, or avoid mentioning, prior or future use of the internet and how GPs respond and/or refer to the internet themselves, (3) To explore patients' perspectives on their consultation and reflect on how easy or otherwise it was to discuss prior and planned use of the internet as well as their views about, and experiences of, GPs looking up material online during the consultation and GP recommendations for the use of online material, (4) To explore GPs' perspectives on the introduction/management of material from the internet by patients in the consultation and their views about, and experiences of, looking up material online during the consultation and recommendations for patients future use, and (5) To synthesise the data from objectives 2,3 and 4 to provide (i) examples of effective consultation practice for GPs, and (ii) guidance for patients on raising the topic of the internet with their GP.

METHODS AND STUDY DESIGN

The methodological approach for the HaRI study builds upon a previous study of doctor-patient communication about medicines in general practice.[8] It consists of three elements: pre-consultation questionnaires, video-recorded consultations, and semi-structured interviews with GPs and patients. We will recruit 10 GPs working in practices situated in Southeast England, purposively sampled for practice size and whether they are a training practice to capture different patient populations and GP communication behaviours. We will aim to recruit an equal number of male and female GPs, representing a variety of ages and ethnicities. To maximise cost efficiency of fieldwork up to two GPs from a given practice may be involved in the study. We will draw on our local Primary Care Research Networks and Noclor research support, a research service which supports projects based in northern London, to assist with the identification of suitable practices and recruitment of GPs.

Pre-consultation questionnaires and video-recorded consultations

We aim to collect 300 pre-consultation questionnaires and associated video-recorded consultations (30 consultations per GP). Recent research places recruitment rates for consultation video-recording at 79%,[9] indicating reasonable acceptability. Patients will be alerted to the study either when booking their appointment by telephone, or by GP reception staff upon presentation at their GP surgery. In both cases, they will be handed a brief information sheet about the study when they check in for their appointment with their GP. This will alert a HaRI Research Associate (RA) in the waiting room that a

For peer review only - http://bmjopen.bmj.com/site/about/guidelines.xhtml

potential participant has arrived. The RA will approach the patient to distribute the Patient Information Sheet and discuss the study. Patients who agree to take part will provide informed consent via a paper consent form. Those who decline to take part will be logged, along with the reason if provided. Those who agree to take part will be given a green sheet of paper with a unique participant number printed on the front of it, which indicates to the GP to record the consultation. Those who decline to take part will be provided with a red sheet of paper, communicating to the GP that the patient has not consented to being recorded.

Each consented participant will be invited to complete the pre-consultation questionnaire before their consultation. As the questionnaire is very brief, impact on the flow of clinical practice is expected to be minimal. The questionnaire will focus on patients' use of information sources before the consultation (deliberately not focusing on the internet so as to avoid drawing attention to our particular interest and thereby avoiding a priming effect). Participants will be invited to give consent to be contacted for a semi-structured interview following their consultation. The consent forms provide participants with the option to consent to the recordings and forms to be stored in the University College London (UCL) data archive for use in future research, subject to an appropriate protocol and ethical approval.

The pre-consultation data will largely be collected via tablets using REDCap (Research Electronic Data Capture). This method may be problematic for people unfamiliar with tablet computers, or for those who have impaired fine motor control. The researcher will assist and provide paper copies as necessary, which will then be manually entered into REDCap by the RA following data collection.

Semi-structured patient and GP interviews

Our sample of up to 300 consultations and associated questionnaires will provide maximum variation from which to purposively select up to 30 patients (approximately 3 per GP) for semi-structured interviews, aiming for a spread of patient characteristics and experiences. Patients will be selected to reflect a wide range of socio-demographic characteristics and use and reference to internet-derived health information. On the latter, the interview participants will be selected to reflect (i) instances in which patients report consulting the internet before their GP consultation and this is raised in the consultation, (ii) instances in which patients report consulting the internet before their GP consultation and this is NOT raised in the consultation, (iii) instances in which the patient refers to the internet in the consultation and this is not reported in the pre-consultation questionnaire (iv) instances in which the GP uses the internet or raises internet use during the consultation. Interviews will explore patient accounts of whether they accessed information on their health issue prior to coming in to see their GP, including such sources as magazine and/or newspaper articles, television programs, or advice from friends and family. We will also explore whether they sought online health information before the consultation, perceptions of GP usage of internet resources during consultations, and discussions of internet-derived health information in the recorded consultation.

In addition to patient interviews, we will interview all 10 participating GPs. This will enable the research team to access GPs' views of patient internet usage to access health information, their own usage of internet resources during consultations, and their perceptions of the discussions with patients on online health information. All interviews will be arranged at a time and place convenient to the participating patient or GP. It will be stressed they are free to withhold response to interview questions or to terminate the interview if they wish. Audio interview data will be saved on an encrypted hard drive and transferred into the UCL data safe haven at the first opportunity.

For peer review only - http://bmjopen.bmj.com/site/about/guidelines.xhtml

Ethical considerations

Ethical approval was granted by the London – Camden & Kings Cross Research Ethics Committee on 8 August 2016. We will include consultations with children and those in which a third party aids an adult patient either due to a language barrier or to provide support, so long as the patient is judged by the researcher to be able to give informed consent. Separate consent forms and Patient Information Sheets for children aged 4-10 and 11-17, as well as for adult companions to patients will be utilised. We will exclude potential respondents unable to consent due to cognitive impairment. Names will be recorded on consent forms and contact details will be collected if participants opt to receive a summary of the findings at the end of the project. These will be kept in a locked filing cabinet, accessible only by the research team. Contact details will be shredded after the reports are sent. Identifying details of participants will be removed at the point of transcription. Video data remains identifiable but will only be seen outside of the project team following explicit informed consent from patients and GPs. The consent forms for the pre-consultation questionnaires and consultation video-records provide participants with an option for their data to be stored in the UCL data archive to allow their data to be used in future research, subject to an appropriate protocol and ethical approval.

The Research Electronic Data Capture (REDCap) platform, which will be used to collect the pre-consultation questionnaires, is a secure, web-based application hosted by UCL designed to enable responses to be automatically uploaded into UCL's data safe haven to maximise data security. The data safe haven has been certified to the ISO 27001 information security standard and conforms to the National Health Service Information Governance Toolkit. It was created using a 'walled garden' approach, where the data is stored, processed and managed within the security of the system, avoiding the complexity of assured end-point encryption. A file transfer mechanism enables information to be transferred into the data safe haven simply and securely. Data from pre-consultation questionnaires will be analysed in the data safe haven. As the data safe haven is not currently able to manage video files, the video-recorded data will be stored on an encrypted hard drive.

Requesting the completion of pre-consultation questionnaires and recording of consultations is potentially intrusive. It will be made clear that the decision to participate or decline will not affect clinical care and that patients can withdraw consent up to the time all the data are collected at their GP's surgery. Patients will be given contact details for the research team, including an email address so they can withdraw without having to speak to anyone. GPs will also be made aware that they are free to decline participation and to withdraw consent up until data collection is completed at their site.

Patient and public involvement

As a team we have extensive experience of working with Patient and Public Involvement (PPI) representatives. Two PPI representatives have assisted the project team in helping shape the research proposal, with particular advice given on the importance and relevance of the research question and the acceptability of the research design for patients. They will also contribute to the design of data collection tools (including the pre-consultation survey and interview topic guides), participant information sheets, and consent forms to ensure comprehensibility. PPI representatives will be asked to comment on the coding scheme for the analysis and on the interpretation of the data

For peer review only - http://bmjopen.bmj.com/site/about/guidelines.xhtml

ANALYSIS

Descriptive statistics will be conducted on the data yielded by the pre-consultation questionnaires, giving a demographic overview of our sample. We will utilise conversation analysis (CA), a well-established micro analytic approach for the analysis of social interaction,[9] to analyse vocal and other visible conduct between GPs and patients during consultations. We will screen the data to build collections of cases where patients introduce prior use of the internet to access health information, and where the GP uses or mentions the internet. Cases will be transcribed in detail, using the Jeffersonian transcription system to capture not only what participants say but how they say it, as well as overlap and gaps in talk[10] to facilitate detailed interactional analysis. Interview data will be analysed using thematic analysis [11], and will focus on patients' and GPs' accounts of use of the internet to access health information both in and outside of the consultation. We will follow standard approaches to thematic analysis including familiarisation with data, generating initial codes, searching for and reviewing themes, and defining and naming themes,[11] thereby safeguarding rigour and avoiding premature formation of themes.

The results will be disseminated via academic routes as well as via professional magazines aimed at GPs and the wider media. We will also seek PPI advice on how and where to disseminate our findings beyond peer-reviewed articles, in particular the development of guidance to support patients wishing to discuss health information derived from the internet in consultations.

DISCUSSION

This project seeks to investigate a longstanding source of potential miscommunication in general practice; namely discussion in the consultation of the use of the internet. Online health discussions in medical consultations has been publicized recently by Professor Helen Stokes-Lampard, Chair of the Royal College of General Practitioners (RCGP) who reported that 80% of her consultations are with patients who have already searched online for a diagnosis.[12] If this is indeed a major source of trouble then we need to describe and delineate the interactional consequences by collecting and analyzing consultation recordings, and to improve the evidence base on GP and patient views of online health information. Communication strategies that aid smooth interaction in relation to discussion of the internet can then be identified. These can form the basis of guidance for GPs on how to implement these strategies as well as joined up guidance for patients on how to effectively raise the topic of the internet with their GP.

ACKNOWLEDGMENTS

The Harnessing Resources from the Internet (HaRI) project is funded by the National Institute for Health Research School for Primary Care Research (NIHR SPCR). The views expressed are those of the authors and not necessarily those of the NHS, the NIHR or the Department of Health. We wish to acknowledge patients and GPs for their support of the project, as well as practice staff for facilitating data collection. We also thank Tyrone Curtis and James Speed for assisting with data collection and management, and Charles Prince and Jonathon Benson for their contribution as PPIs.

FUNDING

This study is supported by a grant from the School of Primary Care scheme. The research costs for the study have been supported by the National Institute for Health Research, School of Primary Care,

For peer review only - http://bmjopen.bmj.com/site/about/guidelines.xhtml

awarded £267,571, November 2015. NHS costs are covered via the Local Clinical Research Network. CP is part funded by NIHR CLAHRC Wessex.

CONTRIBUTORS

FS conceptualised the study and developed the study design with the other co-investigators. MS and LH contributed to the development of study design, and collected and analysed data. FS, MS, LH, HA, RB, GL, EM, CP, and SZ read, edited, and approved the final version.

COMPETING INTERESTS

None declared.

REFERENCES

1. Ziebland S, Wyke S. Health and illness in a connected world: How might sharing experiences on the Internet affect people's health? The Milbank Quarterly 2012;**90**(2):219-49.
2. Bloor M, Horobin G. Conflict and conflict resolution in doctor-patient interactions. In: Cox C, Mead A, eds. A sociology of medical practice. London, UK: Collier-MacMillan, 1975.
3. Stevenson F, Kerr C, Murray E, et al. Information from the internet and the doctor-patient relationship: The patient perspective - a qualitative study BMC Fam Pract 2007;**8**(47).
4. Hay M, Cadigan R, Khanna D, et al. Prepared patients: Internet information seeking by new rheumatology patients. Arthritis & Rheumatism 2008;**59**(4):575-82.
5. Chiu Y. Probing, impelling, but not offending doctors: the role of the internet as an information source for patients' interactions with doctors. Qualitative Health Research 2011;**21**(12):1658-66.
6. Bowes P, Stevenson F, Ahluwalia S, et al. 'I need her to be a doctor': Patients' experiences of presenting health information from the internet in GP consultations. British Journal of General Practice 2012;**62**(604):e732-8.
7. Ahluwalia S, Murray E, Stevenson F, et al. 'A heartbeat moment': Qualitative study of GP views of patients bringing health information from the internet to a consultation. British Journal of General Practice 2010;**60**(571):88-94.
8. Stevenson F, Barry C, Britten N, et al. Doctor-patient communication about drugs: The evidence for shared decision-making. Social Science & Medicine 2000;**50**(6):829-40.
9. Drew P, Chatwin J, Collins S. Conversation analysis: A method for research into interactions between patients and health-care professionals. Health Expectations 2001;**4**(1):58-70.
10. Jefferson G. Glossary of transcript symbols with an introduction. In: Lerner G, ed. Conversation analysis: Studies from the first generation. Amsterdam & Philadelphia: John Benjamins Publishing Company, 2004.
11. Braun V, Clarke V. Using thematic analysis in psychology. Qualitative Research in Psychology 2006;**3**(2):77-101.
12. Wickware C. 'Dr Google enters 80% of my consultations', warns RCGP chair. *Pulse* 2017 4 October 2017.

For peer review only

For peer review only - http://bmjopen.bmj.com/site/about/guidelines.xhtml

BMJ Open

Protocol paper for the 'Harnessing resources from the internet to maximise outcomes from GP consultations (HaRI)' study: A mixed qualitative-methods study

Journal:	*BMJ Open*
Manuscript ID	bmjopen-2018-024188.R1
Article Type:	Protocol
Date Submitted by the Author:	13-Jun-2018
Complete List of Authors:	Seguin, Maureen; University College London, eHealth Unit, Primary Care and Population Health; London School of Hygiene and Tropical Medicine, Centre for Global Chronic Conditions<br>Hall, Laura; University College London, eHealth Unit, Primary Care and Population Health<br>Atherton, Helen; University of Warwick, Unit of Academic Primary Care, Warwick Medical School<br>Barnes, Rebecca; University of Bristol, Centre for Academic Primary Care, Population Health Sciences, Bristol Medical School<br>Leydon, Geraldine; Southampton University, Primary Care and Population Science, Faculty of Medicine<br>Murray, Elizabeth; University College London, eHealth Unit, Primary Care and Population Health<br>Pope, Catherine; University of Southampton, Health Sciences<br>Ziebland, Sue; University of Oxford, Nuffield Department of Primary Care Health Sciences<br>Stevenson, Fiona; University College London, eHealth Unit, Primary Care and Population Health
**Primary Subject Heading**:	General practice / Family practice
Secondary Subject Heading:	Communication, General practice / Family practice, Health services research, Qualitative research, Sociology
Keywords:	Primary Health Care, General Practice Consultations, Family Practice, Physician-patient relations, Video-recording, Conversation analysis

SCHOLARONE™
Manuscripts

Title: Protocol paper for the 'Harnessing resources from the internet to maximise outcomes from GP consultations (HaRI)' study: A mixed qualitative-methods study

Corresponding author:

Seguin, Maureen, eHealth Unit, Primary Care and Population Health, University College London, London, UK, and Centre for Global Chronic Conditions, London School of Hygiene and Tropical Medicine, London, UK. m.seguin@ucl.ac.uk

Full names, departments, institutions, city and country of all co-authors:

Hall, Laura, eHealth Unit, Primary Care and Population Health, University College London, London, UK

Atherton, Helen, Unit of Academic Primary Care, Warwick Medical School, University of Warwick, Coventry, UK

Barnes, Rebecca, Centre for Academic Primary Care, Population Health Sciences, Bristol Medical School, University of Bristol, UK

Leydon, Geraldine, [insert affiliation] Primary care and population science, Faculty of medicine, Southampton university, Southampton, UK

Murray, Elizabeth, eHealth Unit, Primary Care and Population Health, University College London, London, UK

Pope, Catherine, Health Sciences, University of Southampton

Ziebland, Sue, Nuffield Department of Primary Care Health sciences, University of Oxford

Stevenson, Fiona, eHealth Unit, Primary Care and Population Health, University College London, London, UK

Keywords:

Primary health care, general practice consultations, family practice, physician-patient relations, video recording, conversation analysis

Word count excluding title page, abstract, references, figures and tables: 2929

Reference count: 12

ABBREVIATIONS

CA Conversation analysis, GP General Practitioner, HaRI Harnessing resources from the internet, PI Principle Investigator, RA Research Associate, PIS Participant Information Sheet, PPI Patient and Public Involvement, REC Research Ethics Committee, UCL University College London

ABSTRACT

Introduction: Many patients now turn to the internet as a resource for health-care information and advice. However, patients' use of the internet to manage their health has been positioned as a potential source of strain on the doctor-patient relationship in primary care. The current evidence about what happens when internet-derived health information is introduced during consultations has relied on qualitative data derived from interview or questionnaire studies. The 'Harnessing resources from the

For peer review only - http://bmjopen.bmj.com/site/about/guidelines.xhtml

For peer review only

internet to maximise outcomes from GP consultations (HaRI)' study combines questionnaire, interview and video-recorded consultation data to address this issue more fully.

Methods and Analysis: Three data collection methods are employed: pre-consultation patient questionnaires, video-recorded consultations between GPs and patients, and semi-structured interviews with GPs and patients. We seek to recruit 10 GPs practicing in Southeast England. We aim to collect up to 30 patient questionnaires and video-recorded consultations per GP, yielding up to 300. Up to thirty patients (approximately 3 per participating GP) will be selected for interviews sampled for a wide range of socio-demographic characteristics, and a variety of ways the use of, or information from, the internet was present or absent during their consultation. We will interview all 10 participating GPs about their views of online health information; reflecting on their own usage of online information during consultations and their patients' references to online health information. Descriptive, conversation, and thematic analysis will be used respectively for the patient questionnaires, video-recorded consultations, and interviews.

Ethics and Dissemination: Ethical approval has been granted by the London – Camden & Kings Cross Research Ethics Committee. Alongside journal publications, dissemination activities include the creation of a toolkit to be shared with patients and doctors, to guide discussions of material from the internet in consultations.

STRENGTHS AND LIMITATIONS

- A key strength of the study is the use of mixed qualitative methods used to capture what happens during consultations.
- This study will be the first empirically-based indication of how conversations on online health information occurs in general practice.
- The findings of this study will inform the first ever empirically based toolkit for patients and general practitioners to help guide discussion of online health information, minimizing interactional disruption during consultations.
- We may yield a low number of recorded consults wherein the internet is mentioned, thereby limiting what we can explore and conclude about such conversations.

BACKGROUND

Increased access to the Internet has provided patients with access to previously privileged information such as health information aimed at health professionals, alongside experiential information shared by other patients.[1] These developments have heightened the pre-existing "double bind" for patients, who are supposed to be knowledgeable about health conditions and able to manage their own care, yet defer to medical wisdom in the consultation itself.[2]

Previous research has established that both patients and doctors are aware of the tension experienced by patients resulting from expectations to be both knowledgeable and passive during the consultation.[3] Though patients are making wide use of the internet for health information, many have concerns about sharing this with their doctors, fearing to be seen as challenging medical authority.[4, 5] Those who do initiate discussions about internet-derived health expect to have the information, and their efforts at self-management, taken seriously.[6] Many general practitioners (GPs) report concerns about how to respond appropriately when patients refer to such information in consultations, especially considering the limited time available in typical medical consultations for extended discussions.[7] They may also be

For peer review only - http://bmjopen.bmj.com/site/about/guidelines.xhtml

For peer review only

concerned that discussions about internet-derived health information will encourage patients to 'go online,' which may lead to undue patient anxiety. Unfortunately, even occasional miscommunications between GPs and patients can have serious deleterious effects, including loss of patient trust and breakdown of the doctor-patient relationship.[6]

The existing evidence base on consultation discussions of internet-derived health information relies on self-reports, either from interview or questionnaire studies with patients and/or GPs. However, these approaches are vulnerable to the criticism that self-report post-hoc accounts reflect respondent reconstructed accounts of what happened. Alternatively, consultation recordings provide access to interactional details in consultations which do not rely on accounts from GPs or patients. The 'Harnessing resources from the internet to maximise outcomes from GP consultations' (HaRI) study fills a gap in the research by video-recording consultations, in addition to collecting pre-consultation patient data via questionnaire and retrospective data via GP and patient interviews post-consultation.

The HaRI study aims to establish how material from the internet is managed by GPs and patients in consultations. Our objectives are five-fold: (1) To determine the range of sources of information and advice used by a sample of patients in advance of their appointment, (2) To understand the range of ways in which patients mention, or avoid mentioning, prior or future use of the internet and how GPs respond and/or refer to the internet themselves, (3) To explore patients' perspectives on their consultation and reflect on how easy or otherwise it was to discuss prior and planned use of the internet as well as their views about, and experiences of, GPs looking up material online during the consultation and GP recommendations for the use of online material, (4) To explore GPs' perspectives on the introduction/management of material from the internet by patients in the consultation and their views about, and experiences of, looking up material online during the consultation and recommendations for patients future use, and (5) To synthesise the data from objectives 2,3 and 4 to provide (i) examples of effective consultation practice for GPs, and (ii) guidance for patients on raising the topic of the internet with their GP.

METHODS AND STUDY DESIGN

The methodological approach for the HaRI study builds upon a previous study of doctor-patient communication about medicines in general practice.[8] It consists of three elements: pre-consultation questionnaires, video-recorded consultations, and semi-structured interviews with GPs and patients. We will recruit 10 GPs working in practices situated in Southeast England, purposively sampled for practice size and whether they are a training practice to capture different patient populations and GP communication behaviours. We will aim to recruit an equal number of male and female GPs, representing a variety of ages and ethnicities. To maximise cost efficiency of fieldwork up to two GPs from a given practice may be involved in the study. We will draw on our local Primary Care Research Networks and Noclor research support, a research service which supports projects based in northern London, to assist with the identification of suitable practices and recruitment of GPs.

Pre-consultation questionnaires and video-recorded consultations

We aim to collect 300 pre-consultation questionnaires and associated video-recorded consultations (30 consultations per GP). Recent research places recruitment rates for consultation video-recording at 79%,[9] indicating reasonable acceptability. Patients will be alerted to the study either when booking their appointment by telephone, or by GP reception staff upon presentation at their GP surgery. In both cases, they will be handed a brief information sheet about the study when they check in for their appointment with their GP. This will alert a HaRI Research Associate (RA) in the waiting room that a

For peer review only - http://bmjopen.bmj.com/site/about/guidelines.xhtml

potential participant has arrived. The RA will approach the patient to distribute the Patient Information Sheet and discuss the study. Patients who agree to take part will provide informed consent via a paper consent form. Those who decline to take part will be logged, along with the reason if provided. Those who agree to take part will be given a green sheet of paper with a unique participant number printed on the front of it, which indicates to the GP to record the consultation. Those who decline to take part will be provided with a red sheet of paper, communicating to the GP that the patient has not consented to being recorded.

Each consented participant will be invited to complete the pre-consultation questionnaire before their consultation. As the questionnaire is very brief, impact on the flow of clinical practice is expected to be minimal. The questionnaire will focus on patients' use of information sources before the consultation (deliberately not focusing on the internet so as to avoid drawing attention to our particular interest and thereby avoiding a priming effect). Participants will be invited to give consent to be contacted for a semi-structured interview following their consultation. The consent forms provide participants with the option to consent to the recordings and forms to be stored in the University College London (UCL) data archive for use in future research, subject to an appropriate protocol and ethical approval.

The pre-consultation data will largely be collected via tablets using REDCap (Research Electronic Data Capture). This method may be problematic for people unfamiliar with tablet computers, or for those who have impaired fine motor control. The researcher will assist and provide paper copies as necessary, which will then be manually entered into REDCap by the RA following data collection.

Semi-structured patient and GP interviews

Our sample of up to 300 consultations and associated questionnaires will provide maximum variation from which to purposively select up to 30 patients (approximately 3 per GP) for semi-structured interviews, aiming for a spread of patient characteristics and experiences. Patients will be selected to reflect a wide range of socio-demographic characteristics and use and reference to internet-derived health information. On the latter, the interview participants will be selected to reflect (i) instances in which patients report consulting the internet before their GP consultation and this is raised in the consultation, (ii) instances in which patients report consulting the internet before their GP consultation and this is NOT raised in the consultation, (iii) instances in which the patient refers to the internet in the consultation and this is not reported in the pre-consultation questionnaire (iv) instances in which the GP uses the internet or raises internet use during the consultation. Interviews will explore patient accounts of whether they accessed information on their health issue prior to coming in to see their GP, including such sources as magazine and/or newspaper articles, television programs, or advice from friends and family. We will also explore whether they sought online health information before the consultation, perceptions of GP usage of internet resources during consultations, and discussions of internet-derived health information in the recorded consultation.

In addition to patient interviews, we will interview all 10 participating GPs. This will enable the research team to access GPs' views of patient internet usage to access health information, their own usage of internet resources during consultations, and their perceptions of the discussions with patients on online health information. All interviews will be arranged at a time and place convenient to the participating patient or GP. It will be stressed they are free to withhold response to interview questions or to terminate the interview if they wish. Audio interview data will be saved on an encrypted hard drive and transferred into the UCL data safe haven at the first opportunity.

For peer review only - http://bmjopen.bmj.com/site/about/guidelines.xhtml

Ethical considerations

Ethical approval was granted by the London – Camden & Kings Cross Research Ethics Committee on 8 August 2016. We will include consultations with children and those in which a third party aids an adult patient either due to a language barrier or to provide support, so long as the patient is judged by the researcher to be able to give informed consent. Separate consent forms and Patient Information Sheets for children aged 4-10 and 11-17, as well as for adult companions to patients will be utilised. We will exclude potential respondents unable to consent due to cognitive impairment. Names will be recorded on consent forms and contact details will be collected if participants opt to receive a summary of the findings at the end of the project. These will be kept in a locked filing cabinet, accessible only by the research team. Contact details will be shredded after the reports are sent. Identifying details of participants will be removed at the point of transcription. Video data remains identifiable but will only be seen outside of the project team following explicit informed consent from patients and GPs. The consent forms for the pre-consultation questionnaires and consultation video-records provide participants with an option for their data to be stored in the UCL data archive to allow their data to be used in future research, subject to an appropriate protocol and ethical approval.

The Research Electronic Data Capture (REDCap) platform, which will be used to collect the pre-consultation questionnaires, is a secure, web-based application hosted by UCL designed to enable responses to be automatically uploaded into UCL's data safe haven to maximise data security. The data safe haven has been certified to the ISO 27001 information security standard and conforms to the National Health Service Information Governance Toolkit. It was created using a 'walled garden' approach, where the data is stored, processed and managed within the security of the system, avoiding the complexity of assured end-point encryption. A file transfer mechanism enables information to be transferred into the data safe haven simply and securely. Data from pre-consultation questionnaires will be analysed in the data safe haven. As the data safe haven is not currently able to manage video files, the video-recorded data will be stored on an encrypted hard drive.

Requesting the completion of pre-consultation questionnaires and recording of consultations is potentially intrusive. It will be made clear that the decision to participate or decline will not affect clinical care and that patients can withdraw consent up to the time all the data are collected at their GP's surgery. Patients will be given contact details for the research team, including an email address so they can withdraw without having to speak to anyone. GPs will also be made aware that they are free to decline participation and to withdraw consent up until data collection is completed at their site.

Patient and public involvement

As a team we have extensive experience of working with Patient and Public Involvement (PPI) representatives. Two PPI representatives have assisted the project team in helping shape the research proposal, with particular advice given on the importance and relevance of the research question and the acceptability of the research design for patients. They will also contribute to the design of data collection tools (including the pre-consultation survey and interview topic guides), participant information sheets, and consent forms to ensure comprehensibility. PPI representatives will be asked to comment on the coding scheme for the analysis and on the interpretation of the data

For peer review only - http://bmjopen.bmj.com/site/about/guidelines.xhtml

For peer review only

ANALYSIS

Descriptive statistics will be conducted on the data yielded by the pre-consultation questionnaires, giving a demographic overview of our sample. We will utilise conversation analysis (CA), a well-established micro analytic approach for the analysis of social interaction,[9] to analyse vocal and other visible conduct between GPs and patients during consultations. We will screen the data to build collections of cases where patients introduce prior use of the internet to access health information, and where the GP uses or mentions the internet. Cases will be transcribed in detail, using the Jeffersonian transcription system to capture not only what participants say but how they say it, as well as overlap and gaps in talk[10] to facilitate detailed interactional analysis. Interview data will be analysed using thematic analysis [11], and will focus on patients' and GPs' accounts of use of the internet to access health information both in and outside of the consultation. We will follow standard approaches to thematic analysis including familiarisation with data, generating initial codes, searching for and reviewing themes, and defining and naming themes,[11] thereby safeguarding rigour and avoiding premature formation of themes.

The results will be disseminated via academic routes as well as via professional magazines aimed at GPs and the wider media. We will also seek PPI advice on how and where to disseminate our findings beyond peer-reviewed articles, in particular the development of guidance to support patients wishing to discuss health information derived from the internet in consultations.

DISCUSSION

This project seeks to investigate a longstanding source of potential miscommunication in general practice; namely discussion in the consultation of the use of the internet. Online health discussions in medical consultations has been publicized recently by Professor Helen Stokes-Lampard, Chair of the Royal College of General Practitioners (RCGP) who reported that 80% of her consultations are with patients who have already searched online for a diagnosis.[12] If this is indeed a major source of trouble then we need to describe and delineate the interactional consequences by collecting and analyzing consultation recordings, and to improve the evidence base on GP and patient views of online health information. Communication strategies that aid smooth interaction in relation to discussion of the internet can then be identified. These can form the basis of guidance for GPs on how to implement these strategies as well as joined up guidance for patients on how to effectively raise the topic of the internet with their GP.

ACKNOWLEDGMENTS

The Harnessing Resources from the Internet (HaRI) project is funded by the National Institute for Health Research School for Primary Care Research (NIHR SPCR). The views expressed are those of the authors and not necessarily those of the NHS, the NIHR or the Department of Health. We wish to acknowledge patients and GPs for their support of the project, as well as practice staff for facilitating data collection. We also thank Tyrone Curtis and James Speed for assisting with data collection and management, and Charles Prince and Jonathon Benson for their contribution as PPIs.

FUNDING

This study was funded by the National Institute for Health Research School of Primary Care Research. The views expressed are those of the authors and not necessarily those of the NIHR, the NHS or the

For peer review only - http://bmjopen.bmj.com/site/about/guidelines.xhtml

Department of Health. NHS costs are covered via the Local Clinical Research Network. CP is part funded by NIHR CLAHRC Wessex.

CONTRIBUTORS

FS conceptualised the study and developed the study design with the other co-investigators. MS and LH contributed to the development of study design, and collected and analysed data. FS, MS, LH, HA, RB, GL, EM, CP, and SZ read, edited, and approved the final version.

COMPETING INTERESTS

None declared.

REFERENCES

1. Ziebland S, Wyke S. Health and illness in a connected world: How might sharing experiences on the Internet affect people's health? The Milbank Quarterly 2012;**90**(2):219-49.
2. Bloor M, Horobin G. Conflict and conflict resolution in doctor-patient interactions. In: Cox C, Mead A, eds. A sociology of medical practice. London, UK: Collier-MacMillan, 1975.
3. Stevenson F, Kerr C, Murray E, et al. Information from the internet and the doctor-patient relationship: The patient perspective - a qualitative study BMC Fam Pract 2007;**8**(47).
4. Hay M, Cadigan R, Khanna D, et al. Prepared patients: Internet information seeking by new rheumatology patients. Arthritis & Rheumatism 2008;**59**(4):575-82.
5. Chiu Y. Probing, impelling, but not offending doctors: the role of the internet as an information source for patients' interactions with doctors. Qualitative Health Research 2011;**21**(12):1658-66.
6. Bowes P, Stevenson F, Ahluwalia S, et al. 'I need her to be a doctor': Patients' experiences of presenting health information from the internet in GP consultations. British Journal of General Practice 2012;**62**(604):e732-8.
7. Ahluwalia S, Murray E, Stevenson F, et al. 'A heartbeat moment': Qualitative study of GP views of patients bringing health information from the internet to a consultation. British Journal of General Practice 2010;**60**(571):88-94.
8. Stevenson F, Barry C, Britten N, et al. Doctor-patient communication about drugs: The evidence for shared decision-making. Social Science & Medicine 2000;**50**(6):829-40.
9. Drew P, Chatwin J, Collins S. Conversation analysis: A method for research into interactions between patients and health-care professionals. Health Expectations 2001;**4**(1):58-70.
10. Jefferson G. Glossary of transcript symbols with an introduction. In: Lerner G, ed. Conversation analysis: Studies from the first generation. Amsterdam & Philadelphia: John Benjamins Publishing Company, 2004.
11. Braun V, Clarke V. Using thematic analysis in psychology. Qualitative Research in Psychology 2006;**3**(2):77-101.
12. Wickware C. 'Dr Google enters 80% of my consultations', warns RCGP chair. *Pulse* 2017 4 October 2017.

For peer review only

For peer review only - http://bmjopen.bmj.com/site/about/guidelines.xhtml
