# Supplementary material for: Protocol paper for the ‘Harnessing resources from the internet to maximise outcomes from GP consultations (HaRI)’ study: a mixed qualitative methods study
Source: BMJ Open. 2018 Aug 10;8(8):e024188. doi: 10.1136/bmjopen-2018-024188 (PMC6089307; doi:10.1136/bmjopen-2018-024188)
Supplement: Reviewer comments [file bmjopen-2018-024188.reviewer_comments.pdf]

PEER REVIEW HISTORY

BMJ Open publishes all reviews undertaken for accepted manuscripts. Reviewers are asked to complete a checklist review form (**http://bmjopen.bmj.com/site/about/resources/checklist.pdf**) and are provided with free text boxes to elaborate on their assessment. These free text comments are reproduced below.

ARTICLE DETAILS

**TITLE (PROVISIONAL)**	Protocol paper for the ‘Harnessing resources from the internet to maximise outcomes from GP consultations (HaRI)’ study: A mixed qualitative-methods study
**AUTHORS**	Seguin, Maureen; Hall, Laura; Atherton, Helen; Barnes, Rebecca; Leydon, Geraldine; Murray, Elizabeth; Pope, Catherine; Ziebland, Sue; Stevenson, Fiona

This article was not externally reviewed at BMJ Open. Protocols that have been independently assessed prior to submission to BMJ Open are usually fast-tracked to publication on the grounds that further substantial changes will not be possible. This independent assessment will usually be external, independent review for both a substantial grant award from a non-commercial or government funder and ethics approval.
